# Children’s state anxiety before MRI scanning and resting state functional connectivity in large scale brain networks

**DOI:** 10.1038/s41598-025-34410-8

**Published:** 2026-01-16

**Authors:** Purnima Qamar, Dana E. Díaz, Brenda E. Benson, Daniel S. Pine, Peter A. Kirk, Kalina J. Michalska

**Affiliations:** 1 Emotion and Development Branch, National Institute of Mental Health, Bethesda, MD USA 15K North Drive, 20892-MSC; 2 Columbia University Irving Medical Center, New York, NY USA; 3 University of California, Riverside, Riverside, CA USA; 4 University of California, Los Angeles, Los Angeles, CA USA

**Keywords:** Pediatric anxiety, Resting-state functional connectivity, Default mode network, Salience network, Community samples, Pre-scanning state anxiety, Neuroscience, Psychology

## Abstract

**Introduction:**

Most resting-state functional connectivity (rs-FC) research does not consider the participant’s subjective state during magnetic resonance imaging (MRI). Heightened anxiety before an MRI (“pre-scanning state anxiety”) may influence rs-FC and complicate interpretation of individual differences, particularly in underrepresented groups whose scanning experiences may differ from typical research samples.

**Methods:**

We assessed associations between pre-scanning state anxiety and rs-FC within and between the default mode network (DMN) and salience network in a trait-anxious community sample of Latina girls (8–13 years) and a companion sample of treatment-seeking and healthy youth (8–18 years) of predominantly non-Latinx background. A constrained network-based statistical approach calculated the average of un-thresholded correlation coefficients from edge-level partial Spearman correlations to produce network-level measures (7 cortical + 1 subcortical). This approach is “constrained” in that analyses operate at the spatial scale of functional networks, rather than individual edges, to increase statistical power. Statistics were compared against a permutation-based null distribution to assess significance (Bonferroni corrected *p* < 0.00139).

**Results:**

Reduced rs-FC within the DMN (*r* = − 0.32, *p* < 0.00139) was associated with pre-scanning state anxiety in the community sample, but did not replicate in our companion sample.

**Discussion:**

Pre-scanning state anxiety is associated with rs-FC within the DMN, but only among a trait-anxious community sample. Individual differences in MRI scanning experiences may be associated with rs-FC, but sample characteristics and replication should be considered.

**Supplementary Information:**

The online version contains supplementary material available at 10.1038/s41598-025-34410-8.

## Introduction

Greater sample diversity is needed in developmental neuroimaging research^1,2^. Recent work has highlighted barriers to participation, including medical mistrust stemming from adverse historical experiences with scientific research^3,4^, which may influence how research procedures are experienced. Such concerns may be particularly relevant for functional magnetic resonance imaging (fMRI), where the scanning environment itself can be stressful. Heightened anxiety before an MRI procedure (i.e., pre-scanning state anxiety), common in ethnically diverse samples^5^, may be uniquely associated with individual differences in brain activity during MRI^6^. Understanding associations between pre-scanning state anxiety and resting-state functional connectivity (rs-FC) can help researchers interpret individual differences in brain activity, particularly in underrepresented groups whose subjective scanning experiences may differ from those of predominantly non-Latinx white samples typically studied. Clarifying these associations may also aid future research in disentangling whether cross-sample differences in rs-FC reflect neurobiological variation or differences in emotional states during scanning. To address these issues, the current study assessed associations between pre-scanning state anxiety and rs-FC in two independent samples: a community sample of Latina girls (8–13 years) and a predominantly non-Latinx white companion sample of typically-developing youth and youth seeking treatment for one or more anxiety disorders (8–18 years).

Although rs-FC is conceptualized as measuring ‘intrinsic connectivity’, in-scanner experiences undoubtedly influence rs-FC^7,8^. The majority of work on anxiety and rs-FC does not consider the subjective state of the participant during scanning^9^. State and trait anxiety tend to strongly correlate; thus, dissociating them is challenging. Both share common^11^ and distinct neural circuits^10^, yet have not been conclusively mapped. Understanding which circuits differentially associate with state vs. trait anxiety may provide insight into mechanisms of pathological anxiety^12^. However, in samples that have discomfort or anxiety in the scanning environment, differences in brain activity could be misattributed to trait anxiety rather than scanner-evoked state anxiety. Accordingly, research must conclusively map state-related influences from trait effects and identify the brain regions and networks most susceptible to these factors. This is relevant for garnering clinical insight in samples unfamiliar with academic or medical research settings, who may experience greater anticipatory anxiety during MRI scanning^13^ than samples more familiar with such settings.

Trait anxiety reflects an individual’s general disposition toward experiencing anxious states^42,43^. Clinical anxiety represents a maladaptive form of this disposition, characterized by persistent impairment and behaviors such as avoidance^44,45^. Both trait and clinical anxiety have been linked to altered rs-FC in brain networks associated with affective processing and cognitive control^14,15^, namely the default mode network (DMN) and salience network. The DMN, which includes the medial prefrontal cortex (mPFC), precuneus, and posterior cingulate cortex (PCC)^16^, is typically suppressed during attention-demanding tasks^14,15,17^. However, findings are mixed in clinically anxious samples^18,19^, and little is known about how DMN rs-FC is associated with pre-scanning state anxiety. The salience network, a task-positive network that is typically suppressed during rest^20^, is modulated by arousal and supports the detection of salient events^21,22^. It primarily encompasses the dorsal anterior cingulate cortex (ACC) and anterior insula, with subcortical connections to anxiety-relevant regions such as the amygdala^21^. In anxiety disorders, reduced salience network functional connectivity^23^ has been observed during rest, and this reduction has been linked to diminished cognitive control, elevated fear responses, and biased attention to threat^15,24^.

The current study investigated whether pre-scanning state anxiety, expected to persist into the scanning session, is associated with rs-FC within and between the DMN and the salience network across two independent samples from two sites. The primary sample was a community cohort of Latina girls aged 8–13 years with elevated trait anxiety recruited at the University of California, Riverside (UCR) in Southern California; an underrepresented group in research. The companion sample comprised primarily non-Latinx white, affluent, clinically anxious and healthy female youth aged 8–18 years, collected at the National Institute of Mental Health (NIMH). Drawing on prior evidence that state anxiety uniquely contributes to brain function^6,10^, we preregistered the following hypotheses: (https://osf.io/h96qf*)* heightened pre-scanning anxiety would be associated with (1) increased rs-FC within the salience network; (2) altered rs-FC within the DMN (non-directional prediction given mixed prior findings); and (3) decreased functional connectivity between the salience network and DMN.

## Methods

### Participants and procedures

Analyses were conducted separately across two studies: one study examining girls’ emotional development at UCR and the other testing the neurocognitive and clinical features of anxiety disorders at the NIMH. All participants completed self-report measures followed by an fMRI resting-state scan, where they viewed a white fixation cross on a black screen. All participants were instructed to remain still and keep their eyes open for the duration of the scan at both sites. Following the fMRI resting-state scan, participants self-reported their fear during the scanning session.

## University of California, Riverside

Forty-seven Latina girls aged 8–13 (*M*_*age*_ = 10.02, see Table 1) were recruited through community-based outreach and the UCR Child Participant database for a study on socioemotional development approved by the UCR Institutional Review Board and conducted in accordance with UCR guidelines and regulations. Eligibility required at least 50% Latinx heritage and self-identification as ethnically Latina. Many participants also identified their race as white (85.1%), and a minority identified as multiracial Latina (14.7%). Participants were recruited as a healthy sample; however, post-hoc analyses indicated elevated mean levels of self-reported trait anxiety (see Table 2). Participants were excluded if they had a current psychiatric diagnosis of Tourette’s syndrome, obsessive-compulsive disorder, lifetime history of mania, psychosis, or pervasive developmental disorder, or had metal implants or braces. Parents provided informed consent, and minors provided assent. Girls self-reported on trait anxiety (see *Measures*) in a laboratory setting. Girls reported on additional measures of socioemotional development and psychopathology unrelated to the current study. Participants first reported their pre-scanning state anxiety immediately before scanning in the imaging center (see *Measures*). During the scanning session, participants completed an 8-minute eyes-open fMRI resting-state scan. Following the scan, girls self-reported on their in-scanner fear (see *Measures*).

**Table 1:** UCR and NIMH Sample Demographic Characteristics.

Participants	UCR (*N* = 47)	NIMH (*N* = 48)
Age (M, SD)	10.02 (1.18)	13.62 (2.83)
Age (Range)	8–13	8–18
Female	100%	100%

Race	UCR (*N* = 47)	NIMH (*N* = 48)
White	40 (85.1%)^1^	30 (62.5%)
Black		9 (18.75%)
Asian		3 (6.25%)
Other/Mixed	7 (14.7%)^1^	5 (10.41%)

Ethnicity	UCR (*N* = 47)	NIMH (*N* = 48)
Latina or Hispanic	47 (100%)	3 (6.25%)

^1^All participants in the UCR sample were at least 50% Latina.

**Table 2:** *UCR and NIMH Sample Descriptive Statistics of Study Variables*.

STAI-C State	UCR (*N* = 47)	NIMH (*N* = 48)
Mean (SD)	29.60 (5.42)	30 (5.91)
Median	29	30
Range	20–43	20–48

STAI-C Trait	UCR (*N* = 47)	NIMH (*N* = 48)
Mean (SD)	38.43 (7.48)	30.87 (7.96)
Median	39	20.85
Range	22–54	20–52

STAI-C: State-Trait Anxiety Inventory for Children.

## National Institute of Mental Health

The companion sample was a primarily non-Latinx white, affluent sample of clinically anxious and healthy youth (8–18 years; 100% female) collected at the NIMH. This sample included 48 children and adolescents (60% non-Latina white; 100% female) between the ages of 8–18 years old (*M*_*age*_ = 13.62; See Table 1), recruited through the National Institutes of Health Office of Patient Recruitment and advertisements in the community. Participants were eligible if they were between 8 and 18 years old, had no metal (e.g., braces, implants), had an IQ greater than 70, and could consent to the protocol in English. Both boys and girls were recruited, but as the primary analysis of UCR data focused exclusively on girls, only girls were included in these analyses to enable comparisons between datasets. The study was approved by the NIMH Institutional Review Board and performed in accordance with NIMH guidelines and regulations. Parents signed an informed consent, while minor participants signed an informed assent.

The sample consisted of 28 healthy volunteers and 20 youth with one or more anxiety disorders. Anxious youth were diagnosed through a psychiatric interview of the Kiddie Schedule for Affective Disorders and Schizophrenia (K-SADS)^25^ with a licensed clinician. All participants underwent a medical assessment with a licensed clinician before scanning to screen for ineligibility. Participants self-reported their trait anxiety in a laboratory setting, and self-reported on pre-scanning state anxiety immediately before scanning in the imaging center. This group of participants completed a 6-minute fMRI resting-state scan. Following the scan, participants self-reported on their in-scanner fear (see *Measures*).

## Measures

### Questionnaire data

Parents at both sites indicated their child’s race, ethnicity, and age (See Table 1). Notably, the NIMH sample did not have a Hispanic/Latina racial item and only probed Hispanic/Latina ethnic identity.

At both sites, state and trait anxiety were assessed prior to the scan at the imaging center via youth self-report on the State-Trait Anxiety Inventory for Children (STAIC; See Table 2)^26^. The STAIC comprises a 20-item subscale assessing state anxiety (STAIC-State) and a 20-item subscale assessing trait anxiety (STAIC-Trait).

Additionally, directly following the scan, participants at both sites self-reported their in-scanner experience via the MRI Acceptability scale^42^, a 7-item battery of questions probing in-scanner experiences (e.g., fear, happiness). One item reporting in-scanner fear was used for post-hoc analyses.

### MR images acquisition

### University of California, Riverside

Participants at UCR underwent scanning in a 3T Siemens Prisma with a 32-channel head coil as part of a protocol at the Kids Interaction and NeuroDevelopment (KIND) Lab. The scanner used a single-shot T2*-weighted echo-planar imaging (EPI) with the following parameters: duration = 8 min, 60 slices, flip angle = 75, TE = 30 ms, TR = 800 ms, FOV = 216 mm, acquisition voxel size = 2.4 × 2.4 × 2.4 mm^3^. High-resolution single-shot T1-weighted anatomical images were collected using a standardized magnetization-prepared spoiled gradient-recalled echo sequence: 208 slices, flip angle = 8, TE = 2.72 ms, TR = 2400 ms, FOV = 240 × 256 mm, acquisition voxel size = 0.8 × 0.8 × 0.8 mm^3^.

### National Institute of Mental Health

Imaging data were acquired with one of two General Electric 3T scanners (Signa and Discovery) at the National Institutes of Health Imaging Facility, with a consistent acquisition sequence across scanners. Each scanner used identical single-shot T2*-weighted echo planar imaging (EPI) with the following parameters: 180 volumes, flip angle = 90, TE = 30 ms, TR = 2000 ms, FOV = 192 mm, matrix = 64 × 64, voxel size = 3 × 3 × 4 mm^3^. High-resolution T1-weighted anatomical images were collected using standardized magnetization-prepared spoiled gradient-recalled echo sequence: 124 slices, flip angle = 6, TE = 3.0 ms, TR = 7.8 mm, FOV = 256 mm, acquisition voxel size = 0.86 × 0.86 × 1.2 mm^3^.

### Preprocessing

At each site, individual echo-planar images were preprocessed and denoised with Freesurfer^27^ and AFNI^28^. In brief, this included despiking (3dDespike), slice-time correction, realignment of functional volumes (align_epi_anat.py), registration of functional volumes to anatomy (3dvolreg), spatial transformation to Montreal Neurological Institute (MNI) space^29^, and scaling (3dcalc). Data was then denoised by taking the residual time series from a general linear model with the following regressors: 12 motion parameters, motion censoring (framewise displacement 0.25 mm thresholds), and demeaning/detrending with polynomials (3 polynomials; -polort = 3).

For each participant, activation time series were extracted across 200 cortical regions (as defined using the Schaefer Atlas^30^ and 16 subcortical regions (defined using the Melbourne Subcortex Atlas^31^ from the preprocessed and denoised data. Cortical networks comprise the DMN, salience network, control network, dorsal attention network, limbic network, visual network, and somatomotor networks, defined by the Schaefer 200 parcels and 7 network parcellation^30^. The subcortex network comprises 16 bilateral regions: the amygdala, hippocampus, posterior and anterior thalamus, caudate, nucleus accumbens, globus pallidus, and putamen^31^. Functional connectivity measures were then calculated via Fisher-transformed bivariate correlations between all pairwise combinations of time series with AFNI’s 3dNetCorr function for the DMN and salience networks, followed by exploratory associations with the remaining networks.

### Final sample

Following quality control and preprocessing, 87 children remained in the sample (*N*_*UCR*_ = 42, *N*_*NIMH*_ = 45). Three participants at the NIMH were excluded for failing quality control checks, and 5 participants at UCR were excluded for either failed preprocessing (*n* = 1) or missing pre-scanning state anxiety data (*n* = 4).

### Analyses

Following data collection, hypotheses and analyses were pre-registered on OSF (https://osf.io/h96qf*)* and analyses were conducted with Python^32^ and AFNI^28^. All analyses used two-sided tests with α = 0.05. Analyses were conducted separately for each site due to demographic and socioeconomic differences between samples.

### Group-level analysis

For group-level analyses, partial Spearman correlations were conducted comparing every edge (functional connectivity measure) with pre-scanning state anxiety (STAI-state). Age and trait anxiety (STAI-trait) were included as covariates to help isolate the effects of pre-scanning state anxiety on rs-FC.

A constrained network-based statistical approach^33^ was used. Here, we calculated the average of un-thresholded correlation coefficients from edge-level partial Spearman correlations to produce network-level measures. Statistics were compared against a null distribution based on averaged correlations from a standard 1000 edge-level permutations to assess significance. Primary hypothesis testing focused on within DMN connectivity (*N*_*ROIs*_ = 44; *N*_*edges*_ = 946), within salience network connectivity (*N*_*ROIs*_ = 20; *N*_*edges*_ = 190), and between DMN-salience connectivity (*N*_*ROIs*_ = 64; *N*_*edges*_ = 880) with a threshold of *p* < 0.05.

Exploratory analyses probed the effect of pre-scanning state anxiety on connectivity within and between all eight networks (7 cortical + 1 subcortical). Uncorrected results (*p* < 0.05) are reported below, as well as Bonferroni-corrected results across all 36 within and between-network measures (*p* < 0.00139).

### Post-Hoc analyses

Departing from the pre-registration, we conducted seven post-hoc analyses to assess the robustness of our effects. Across both sites, we (1) re-estimated models without trait anxiety as a covariate to test whether the association with pre-scanning state anxiety remained. In the NIMH sample, we then (2) added a control for the scanner (Signa, Discovery) to evaluate potential scan site effects; (3) constrained the sample to girls aged 8–13 to assess developmental stage influences; (4) constrained the sample to participants whose resting-state scan occurred first in the protocol to address order effects; and (5) computed Pearson correlations between pre-scanning state anxiety and in-scanner fear experiences within each sample. Next, we (6) combined functional connectivity matrices from the full NIMH sample and from UCR participants who consented to data sharing to increase power and generalizability. Finally, to address concerns that hypothesized relationships could be driven by head motion, we (7) tested the association between individual differences in pre-scanning state anxiety and in-scanner head motion (AFNI Euclidean norm of motion parameters).

## Results

**Fig. 1:**
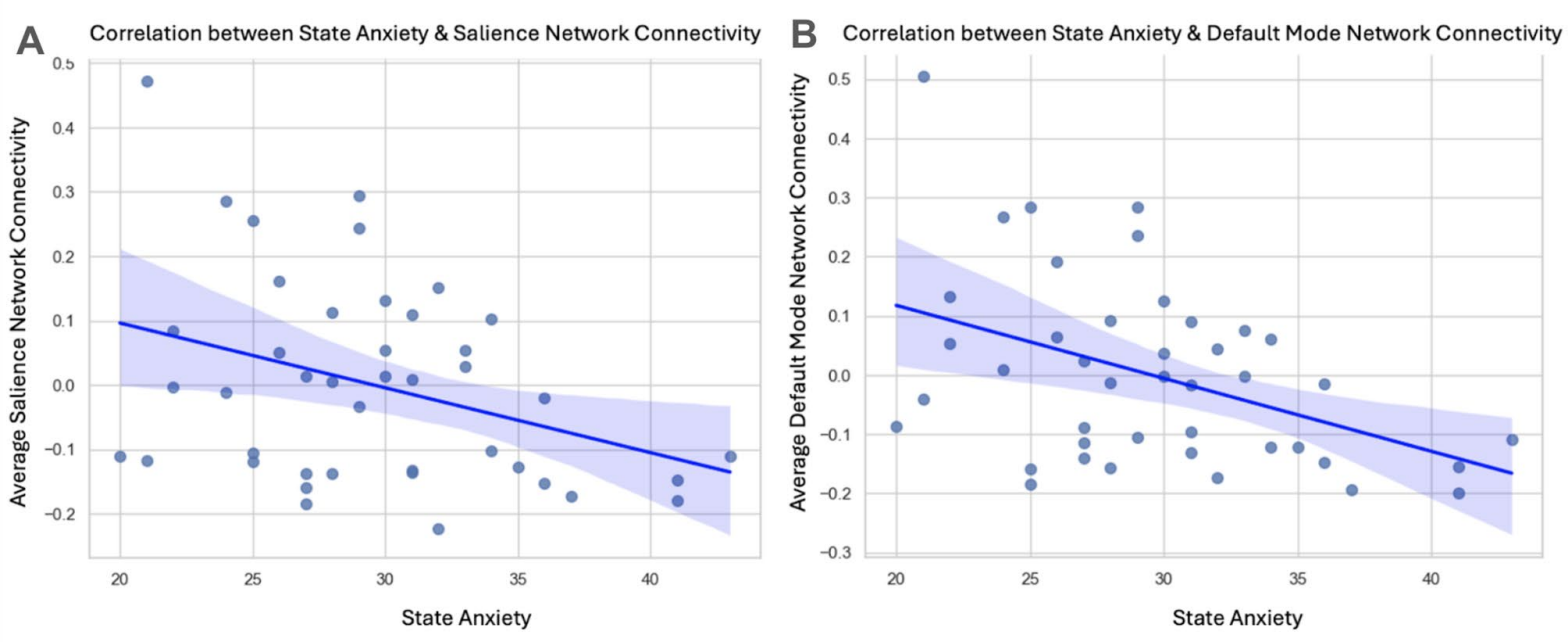
UCR Pre-scanning State Anxiety and Within-Network Connectivity. Graphs display the (non-significant) partial correlations between (**A**) pre-scanning state anxiety and resting-state functional connectivity (rs-FC) within the salience network (*r*(40) = − 0.19; *p* = .096) and a (**B**) significantly negative correlation between pre-scanning state anxiety and rs-FC within the default mode network (DMN), controlling for age and trait anxiety (*r*(40) = − 0.32; *p* = .0001).

**Fig. 2:**
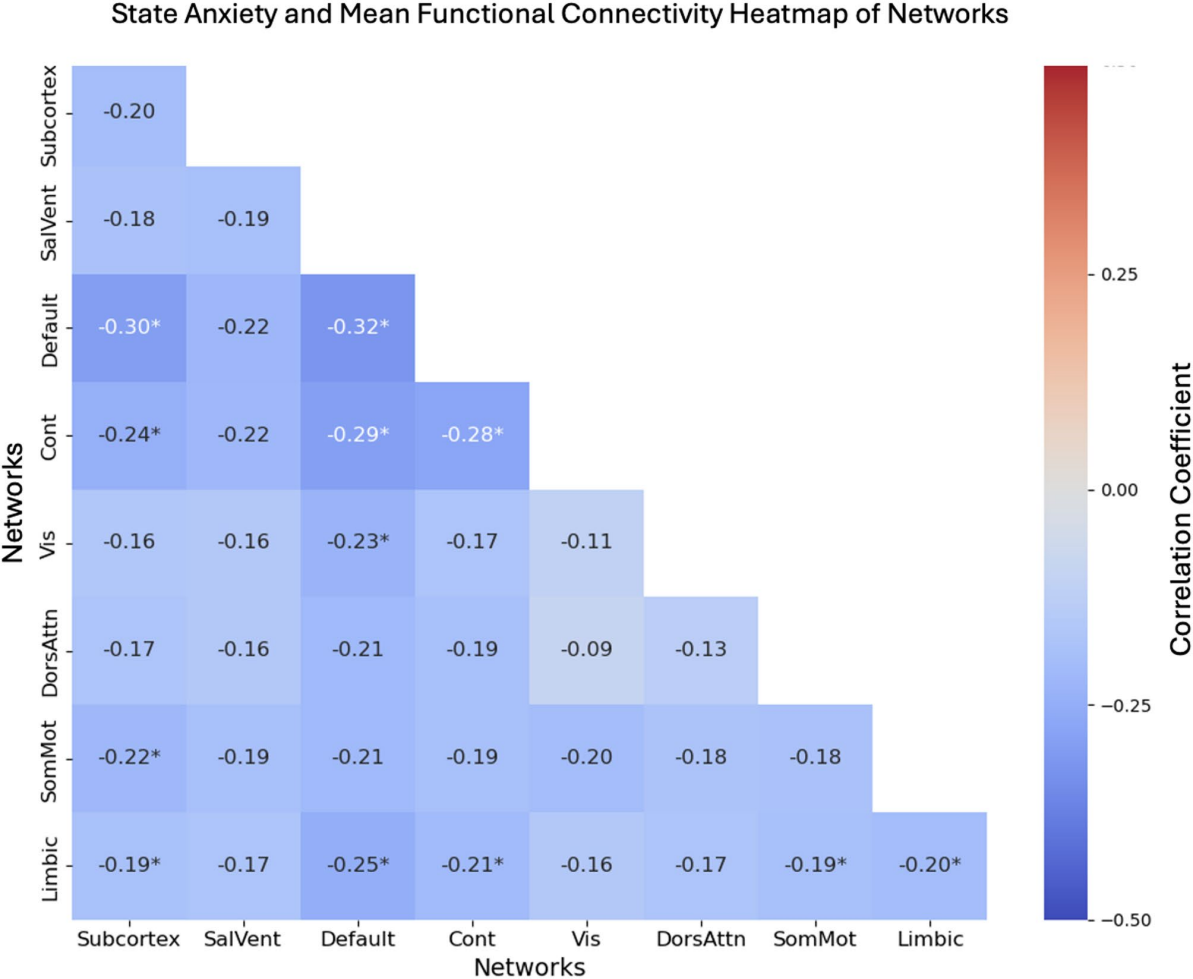
UCR Sample Resting-State Functional Connectivity. The heat map displays partial correlation between pre-scan state anxiety and resting-state functional connectivity (rs-FC) within and between 7 networks. A significant association emerged within the default mode network (DMN) (*p* = .0001). Red color indicated positive connectivity between networks, whereas blue indicates negative connectivity. Subcortex = Subcortical Regions, SalVent = Salience Ventral Attention Network, Default = Default Mode Network, Cont = Control Network, Vis = Visual Network, DorsAttn = Dorsal Attention Network, SomMot = Somatomotor Network, Limbic = Limbic Network. Heatmap was generated using Python’s Seaborn data visualization package (version 0.13.2.; https://seaborn.pydata.org)^50^.

In our hypothesis testing, we observed significantly reduced rs-FC within the DMN as a function of pre-scanning state anxiety (See Fig. 1), controlling for age and trait anxiety, which passed our Bonferroni threshold of *p* < .00139 in the UCR community sample (*p* = .0001). Whole-brain exploratory analyses revealed that pre-scanning state anxiety was associated with reduced within-network rs-FC in the Limbic and Control networks (all *ps* < .05), although these did not pass Bonferroni correction (See Supplement 2A ). Additionally, pre-scanning state anxiety was associated with reduced between-network rs-FC of the Subcortex, DMN, Limbic, Control, Visual, and Somatomotor Networks (Fig. 2), but none passed Bonferroni correction. (See Supplement 2A)

### University of California, Riverside sample

### National Institute of Mental Health sample

**Fig. 3:**
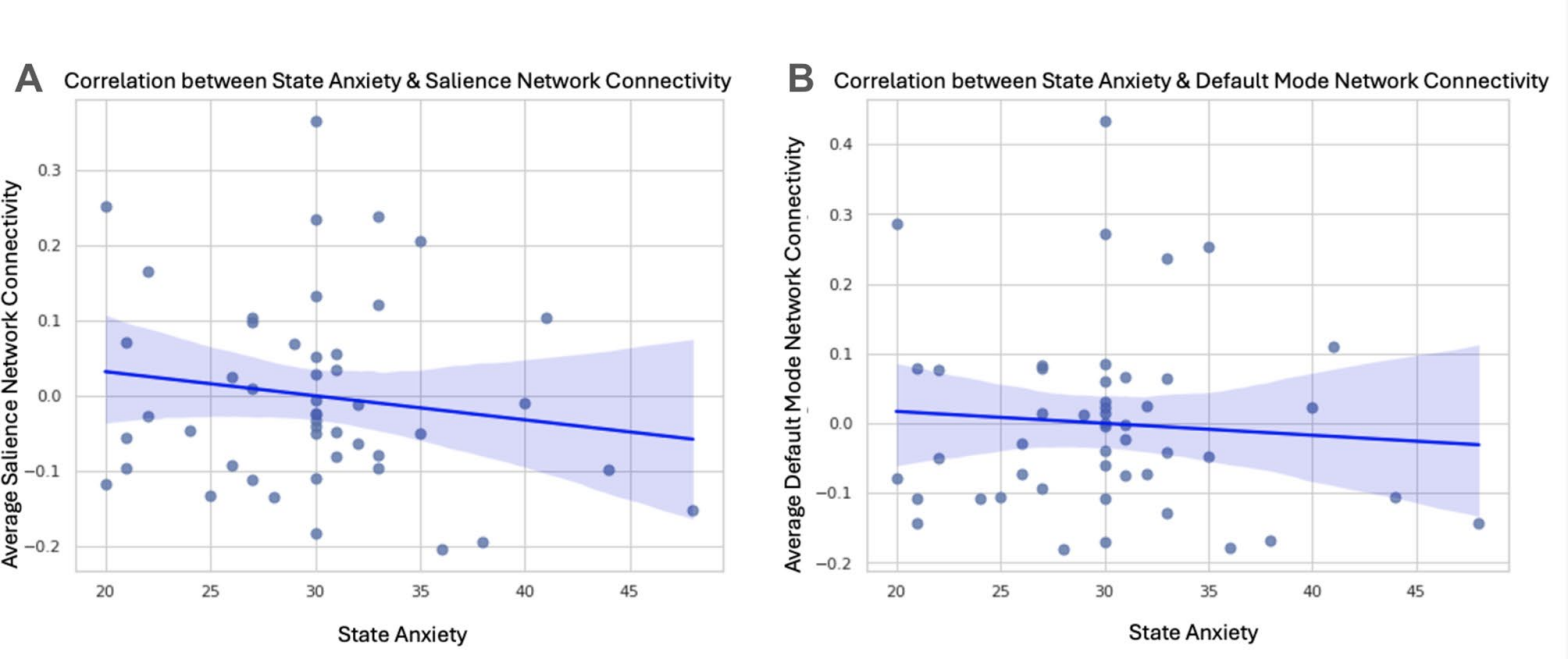
NIMH Pre-scanning State Anxiety and Within-Network Connectivity. Graphs display the (non-significant) correlation between pre-scanning state anxiety and resting-state functional connectivity within the (**A**) salience (*r*(43) = − 0.04) and (**B**) default mode network (DMN) (*r*(43) = − 0.02), controlling for age and trait anxiety (all *ps* < .65).

**Fig. 4:**
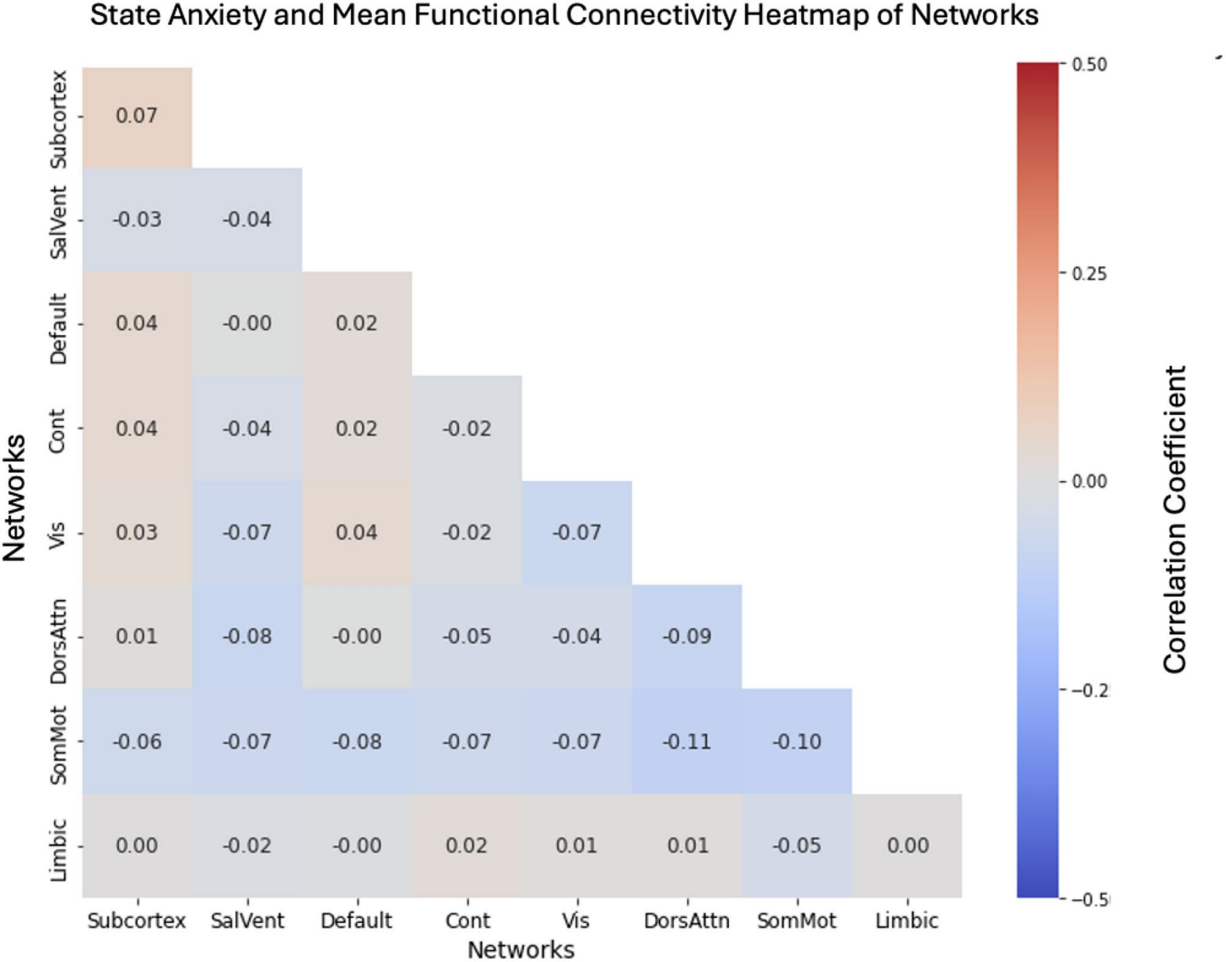
NIMH Sample Resting-State Functional Connectivity. Heat map displays average functional connectivity within and between 7 networks. No significant within or between-network findings emerged (all *ps* > .21). Red color indicates positive connectivity between networks, whereas blue indicates negative connectivity. Subcortex = Subcortical Regions, SalVent = Salience Ventral Attention Network, Default = Default Mode Network, Cont = Control Network, Vis = Visual Network, DorsAttn = Dorsal Attention Network, SomMot = Somatomotor Network, Limbic = Limbic Network. Heatmap was generated using Python’s Seaborn data visualization package (version 0.13.2.; https://seaborn.pydata.org)^50^.

No associations emerged between pre-scanning state anxiety and rs-FC within or between the salience (Fig. 3a) and DMN (Fig. 3b) in the NIMH sample (all *ps* > .22), controlling for age and trait anxiety (See Supplement 2C). Additionally, no associations emerged between pre-scanning state anxiety and whole-brain rs-FC (See Fig. 4). These results did not change when controlling for scanner type or constraining the sample to only children with the resting-state scan at their first MRI scan in the NIMH protocol. Table 2 summarizes the average differences between pre-scanning state anxiety and trait anxiety across both samples.

### Post-Hoc analyses

Following our pre-registered analyses, we conducted post-hoc analyses to examine the stability of our effects. At the UCR site, omitting trait anxiety as a covariate did not change the corrected significance in the DMN (*p* = .0001; see Supplement 2B). However, significant reductions within and between DMN-salience and exploratory network connectivity emerged, but none passed corrections, except for DMN (*p* = .0001; see Supplement 2B). No changes emerged in our NIMH site when omitting trait anxiety as a covariate (see Supplement 2D), controlling for scanner, or constraining the sample to children with the resting-state scan as their first scan in the protocol. However, we did observe an uncorrected increase (*r* = 0.33, *p* = .019) in the DMN-salience network connectivity as a function of state anxiety at the NIMH site when constraining the sample to girls between 8 and 13 (*n* = 20). To probe the validity of our state anxiety measure (STAI-state) as persisting into the scanning session, we conducted Pearson correlations to test the association between pre-scanning state anxiety and in-scanner fear experiences measured through self-report directly following each MRI scan per sample. Here, we observed a significant correlation in the UCR sample (*r =* 0.33, *p* = .03), but missing data resulted in an insufficient sample for analysis of the NIMH sample (valid *N* = 9). All participants in the NIMH sample with viable data self-reported “0” in-scanner fear experiences, suggesting negligible anxiety during scanning. Additionally, combining functional connectivity matrices with the complete NIMH data and UCR participants who consented to public data sharing revealed no significant association within or between the DMN, salience, or DMN-salience networks (all *p*s > .098) (see Supplement 1). Finally, we observed no significant associations between head motion and pre-scanning state anxiety in the NIMH (*r* = − 0.15, *p* = .30) or UCR sample (*r* = 0.15, *p* = .30).

## Discussion

The present study examined associations between pre-scanning state anxiety and resting-state functional connectivity (rs-FC) in a community-based sample of preadolescent Latina girls aged 8–13. To test generalizability, this was followed up with parallel analyses in a companion sample of non-Latinx youth; both typically-developing and treatment-seeking for one or more anxiety disorders (8–18 years). Three key findings emerged. First, in the Latina girls sample, higher pre-scanning state anxiety was associated with decreased rs-FC within the default mode network (DMN); no such association was observed in the companion sample. Second, contrary to predictions, we observed no associations between pre-scanning state anxiety and rs-FC within the salience network or between the salience network and DMN in either sample. Third, in the companion sample, pre-scanning state anxiety was not associated with rs-FC within or between any cortical or subcortical brain networks. These patterns suggest that demographic, socioeconomic, or other experiential factors may introduce heterogeneity in pre-scanning state anxiety, with downstream effects on observed rs-FC. Alternatively, the discrepancy across samples could reflect a false positive. We consider these possibilities in light of the study’s limitations.

The DMN is a task-negative brain network^34,35^ associated with rumination^36^ and interoceptive processing^37^, and is typically suppressed during cognitively demanding tasks^34^. Heightened pre-scanning state anxiety could increase vigilance and situational awareness of the scanning environment; associated with task-positive cognitive demands^38^. Thus, associations between pre-scanning state anxiety and DMN rs-FC may reflect individuals’ experiences with the scanner context. Although children were acclimated to the MRI’s auditory stimuli, physical sensations, and task procedures using a mock scanner before the scanning session, several factors, including the scanner being larger and louder than the mock scanner, and the novelty of the research setting for the community-based sample, may have amplified the association between pre-scanning state anxiety and DMN connectivity^9^. Specifically, unfamiliarity and sensitivity to the scanning environment may have reduced DMN-related processes (e.g., rumination and interoceptive processing), by shifting attention toward hypervigilance and situational awareness. Consistent with this interpretation, pre-scanning state anxiety was associated with in-scanner fear in the UCR sample.

Disruptions in salience network connectivity have been proposed as a possible neural underpinning of anxiety disorders beginning in childhood^39^. Furthermore, increased trait anxiety in adolescents and adults is associated with decreased connectivity between the DMN and salience network^40^. However, we found no support for our hypothesis that pre-scanning state anxiety would be associated with rs-FC within the salience network or between the DMN and salience networks. This contrasts with prior work, which finds pre-scanning state anxiety to be associated with decreased within-network connectivity in the salience network in adults^10,40^. This may reflect a true developmental difference in the association between state anxiety and DMN connectivity. Alternatively, because pre-scanning state and trait anxiety were correlated in the NIMH sample (but not in the UCR sample), controlling for trait anxiety could have attenuated potential associations with state anxiety, although our post-hoc analyses removing trait anxiety as a covariate did not change the pattern of results.

Coupled decreases in DMN, control network, and DMN-control network rs-FC may reflect anxiety-relevant aberrations in the triple network model of anxiety^46,47^. As DMN-control network hypo-connectivity is a feature of anxiety disorders^15^, reduced top-down regulation by the control network may fail to suppress hypervigilance during rest, potentially leading to decreased rs-FC within the DMN. However, as our work controlled for trait anxiety, future work should disentangle associations of pre-scanning state anxiety with DMN-control network rs-FC.

### Limitations

Several limitations should be acknowledged. First, reproducible neuroimaging studies typically require large sample sizes^41^; our modest sample sizes (*N*_*UCR*_ = 42, *N*_*NIMH*_ = 45) limit the generalizability of our results. Results from the UCR sample may represent a false positive, underscoring the need for replication in larger cohorts. Second, demographic, socioeconomic, and situational differences between the UCR and NIMH samples limit direct comparisons. The UCR sample was a community-based group of children from lower socioeconomic whereas the NIMH sample consisted primarily of affluent youth who may have had greater prior exposure to medical and research settings. Race is also a relevant limitation: although the UCR sample consisted entirely of Latina girls, most identified as white Latina, with a smaller proportion identifying as multiracial Latina. Because of the small numbers in each racial subgroup, we could not meaningfully control for or examine racial variation. Third, the UCR sample included only female participants, limiting generalizability; future studies should include both sexes to test for sex-specific effects. Most importantly, meaningful differences in lived experiences may have contributed to variability in prescanning anxiety. For example, limited prior exposure to research and medical settings may have heightened pre-scanning state anxiety in the UCR sample, as suggested by correlations between state anxiety and in-scanner fear. However, it remains unclear to what extent such effects are attributable to any single factor. Future research should directly assess potential contributions, such as medical mistrust before scanning, and test their associations with rs-FC. Evaluating and accounting for participants’ lived experiences in neuroimaging research can be facilitated through mechanisms such as community advisory boards^1,48^. In our own Community and Youth Advisory Board meetings^1^, families in the Inland Empire have reported considerable medical mistrust; while these discussions were not part of the present study’s measures, they motivate incorporating validated mistrust and prior exposure instruments in future work. Finally, subjective scanning experience could be probed beyond self-report using physiological responses (e.g., skin conductance, heart rate) to capture arousal throughout the scanning session. Given evidence that subjective fear can diverge from autonomic arousal in anxiety^49^, concurrent physiological data would provide complementary insight. In the absence of such measures, it is unclear whether pre-scanning state anxiety reflects a sustained affective state across the duration of the resting-state scan.

The NIMH dataset comprised children undergoing the resting-state scan at their first or ninth scan at the NIMH, with the resting-state scan positioned before or after a cognitive task. Not only may there have been residual effects of the cognitive task in some children, but much of the NIMH sample had significantly more prior exposure to the scanning environment than the UCR sample. Thus, the replicability of the UCR finding should be tested in future work with similar samples with similar exposure rates to determine whether effects might be demographic-specific or primarily driven by prior scanning exposure. Future work may also control for previous scanning exposure and aversive reactions during scanning. Additionally, since the reported pre-scanning state anxiety in our samples was moderate, future work may seek to examine associations of pre-scanning state anxiety as rs-FC in samples with greater pre-scanning state anxiety. Finally, given the differences in resting-state scan duration (UCR: 8 min, NIMH: 6 min), future work should compare samples with equivalent acquisition lengths.

In sum, our study finds pre-scanning state anxiety is associated with rs-FC within the DMN, but only among a community sample of preadolescent Latina girls with elevated trait anxiety. These findings suggest that individual differences in MRI scanning experiences relate to brain activity, and that sample demographics and prior exposure to the MRI and research settings should be considered. Future work on brain-behavior relations in these groups may benefit from specific interventions to reduce scanning-related anxiety (e.g., extended mock scanner training). More broadly, developmental neuroscience research with diverse and underrepresented samples should account for the influences of the scanning environment on rs-FC. Replication in larger well-powered samples is needed.

## Supplementary Information

Below is the link to the electronic supplementary material.

Supplementary Material 1

## Data Availability

Data sharing for our NIMH sample analyzed in the current study is not available as the data was collected prior to broad data sharing language being included in 01-M-0192. The data for our UCR sample analyzed during the current study are available in an OSF repository at: https://osf.io/7yujc/files/osfstorage.

## References

[1] La Scala, S., Mullins, J. L., Firat, R. B. & Michalska, K. J. Emotional Learning Research Community Advisory Board, Equity, diversity, and inclusion in developmental neuroscience: Practical lessons from community-based participatory research. *Front. Integr. Nuerosci.* **16**, 1007249 (2023). 10.3389/fnint.2022.1007249 PMC10060815 37007188

[2] Ricard, J. A. et al. Confronting Racially exclusionary practices in the acquisition and analyses of neuroimaging data. *Nat. Neurosci.* **26** (1), 4–11 (2023). 36564545 10.1038/s41593-022-01218-y PMC12884511

[3] El-Galaly, T. C. et al. A lack of diversity, equity, and inclusion in clinical research has direct impact on patient care. *HemaSphere* **7** (3), e842 (2023). 36844176 10.1097/HS9.0000000000000842 PMC9946429

[4] Jaiswal, J. & Halkitis, P. N. Towards a more inclusive and dynamic Understanding of medical mistrust informed by science. *Behav. Med. (Washington D C)*. **45** (2), 79–85 (2019). 10.1080/08964289.2019.1619511 PMC7808310 31343962

[5] Harnett, N. G., Merrill, L. C. & Fani, N. Racial and ethnic socioenvironmental inequity and neuroimaging in psychiatry: a brief review of the past and recommendations for the future. *Neuropsychopharmacology: Official Publication Am. Coll. Neuropsychopharmacol.* **50** (1), 3–15 (2024). 10.1038/s41386-024-01901-7 PMC11526029 38902354

[6] Díaz, D. E., Tseng, W. L. & Michalska, K. J. Pre-scan state anxiety is associated with greater right amygdala-hippocampal response to fearful versus happy faces among trait-anxious Latina girls. *BMC Psychiatry*. **24** (1), 1 (2024). 38167015 10.1186/s12888-023-05403-6 PMC10759434

[7] Finn, E. S. Is it time to put rest to rest? *Trends Cogn. Sci.* **25** (12), 1021–1032 (2021). 34625348 10.1016/j.tics.2021.09.005 PMC8585722

[8] Gonzalez-Castillo, J. et al. In-Scanner Thoughts shape Resting-state Functional Connectivity: how participants rest matters. *bioRxivorg* (2024). 10.1101/2024.06.05.596482

[9] Michalska, K. J., Gardiner, G. & Hughes, B. L. What neuroscience can tell Us about social situations: challenges and opportunities. In: (eds Rauthmann, J. F., Sherman, R. A. & Funder, D. C.) The Oxford Handbook of Psychological Situations. Oxford University Press. (2020).

[10] Saviola, F. et al. Trait and state anxiety are mapped differently in the human brain. *Sci. Rep.* **10** (1), 11112 (2020). 32632158 10.1038/s41598-020-68008-z PMC7338355

[11] Chavanne, A. V. & Robinson, O. J. The overlapping neurobiology of induced and pathological anxiety: A meta-analysis of functional neural activation. *Am. J. Psychiatry*. **178** (2), 156–164 (2021). 33054384 10.1176/appi.ajp.2020.19111153 PMC7116679

[12] Robinson, O. J., Vytal, K., Cornwell, B. R. & Grillon, C. The impact of anxiety upon cognition: perspectives from human threat of shock studies. *Front. Hum. Neurosci.* **7**, 203 (2013). 23730279 10.3389/fnhum.2013.00203 PMC3656338

[13] Forshaw, K. L. et al. Raised anxiety levels among outpatients Preparing to undergo a medical imaging procedure: prevalence and correlates. *J. Am. Coll. Radiology: JACR*. **15** (4), 630–638 (2018). 10.1016/j.jacr.2017.12.030 29503146

[14] Liao, W. et al. Selective aberrant functional connectivity of resting state networks in social anxiety disorder. *NeuroImage* **52** (4), 1549–1558 (2010). 20470894 10.1016/j.neuroimage.2010.05.010

[15] Xu, J. et al. Anxious brain networks: A coordinate-based activation likelihood Estimation meta-analysis of resting-state functional connectivity studies in anxiety. *Neurosci. Biobehav. Rev.* **96**, 21–30 (2019). 30452934 10.1016/j.neubiorev.2018.11.005

[16] Raichle, M. E. The brain’s default mode network. *Annu. Rev. Neurosci.* **38** (1), 433–447 (2015). 25938726 10.1146/annurev-neuro-071013-014030

[17] Whitfield-Gabrieli, S. & Ford, J. M. Default mode network activity and connectivity in psychopathology. *Ann. Rev. Clin. Psychol.* **8** (1), 49–76 (2012). 22224834 10.1146/annurev-clinpsy-032511-143049

[18] Zhao, X. H. et al. Altered default mode network activity in patient with anxiety disorders: an fMRI study. *Eur. J. Radiol.* **63** (3), 373–378 (2007). 17400412 10.1016/j.ejrad.2007.02.006

[19] Zugman, A., Jett, L., Antonacci, C., Winkler, A. M. & Pine, D. S. A systematic review and meta-analysis of resting-state fMRI in anxiety disorders: need for data sharing to move the field forward. *J. Anxiety Disord.* **99** (102773), 102773 (2023). 37741177 10.1016/j.janxdis.2023.102773 PMC10753861

[20] Di, X. & Biswal, B. B. Dynamic brain functional connectivity modulated by resting-state networks. *Brain Struct. Function*. **220** (1), 37–46 (2015). 10.1007/s00429-013-0634-3 PMC3980132 25713839

[21] Seeley, W. W. The salience network: A neural system for perceiving and responding to homeostatic demands. *J. Neuroscience: Official J. Soc. Neurosci.* **39** (50), 9878–9882 (2019). 10.1523/JNEUROSCI.1138-17.2019 PMC6978945 31676604

[22] Xia, C., Touroutoglou, A., Quigley, K. S., Barrett, F., Dickerson, B. C. & L., & Salience network connectivity modulates skin conductance responses in predicting arousal experience. *J. Cogn. Neurosci.* **29** (5), 827–836 (2017). 27991182 10.1162/jocn_a_01087 PMC5690982

[23] Xiong, H., Guo, R. J. & Shi, H. W. Altered default mode network and salience network functional connectivity in patients with generalized anxiety disorders: an ICA-Based Resting‐State fMRI study. *Evidence-Based Complement. Altern. Med.*10.1155/2020/4048916 (2020). 10.1155/2020/4048916 PMC7443230 32855650

[24] Perino, M. T. et al. Whole-Brain Resting-State functional connectivity patterns associated with pediatric anxiety and involuntary attention capture. *Biol. Psychiatry Global Open. Sci.* **1** (3), 229–238 (2021). 10.1016/j.bpsgos.2021.05.007 PMC9417088 36033105

[25] Kaufman, J., Birmaher, B., Brent, D. A., Ryan, N. D. & Rao, U. K-sads-pl. *J. Am. Acad. Child Adolesc. Psychiatry*. **39** (10), 1208 (2000). 11026169 10.1097/00004583-200010000-00002

[26] Spielberger, C. D., Gonzalez-Reigosa, F., Martinez-Urrutia, A., Natalicio, L. F. S. & Natalicio, D. S. The State-Trait anxiety inventory. *Revista Interamericana De Psicología/Interamerican J. Psychol.*10.30849/rip/ijp.v5i3 (1971).

[27] Fischl, B. *FreeSurfer NeuroImage*, **62**(2), 774–781. (2012). 22248573 10.1016/j.neuroimage.2012.01.021 PMC3685476

[28] Cox, R. W. AFNI: software for analysis and visualization of functional magnetic resonance neuroimages. *Comput. Biomed. Res. Int. J.* **29** (3), 162–173 (1996). 10.1006/cbmr.1996.0014 8812068

[29] Mazziotta, J. C., Toga, A. W., Evans, A., Fox, P. & Lancaster, J. A probabilistic atlas of the human brain: theory and rationale for its development. The international consortium for brain mapping (ICBM). *NeuroImage* **2** (2), 89–101 (1995). 9343592 10.1006/nimg.1995.1012

[30] Schaefer, A. et al. Local-global parcellation of the human cerebral cortex from intrinsic functional connectivity MRI. *Cereb. Cortex (New York N Y : 1991)*. **28** (9), 3095–3114 (2018). 10.1093/cercor/bhx179 PMC6095216 28981612

[31] Tian, Y., Margulies, D. S., Breakspear, M. & Zalesky, A. Topographic organization of the human subcortex unveiled with functional connectivity gradients. *Nat. Neurosci.* **23** (11), 1421–1432 (2020). 32989295 10.1038/s41593-020-00711-6

[32] Van Rossum, G. & Drake, F. L. Jr *Python 3 Reference Manual* (Createspace, 2009).

[33] Noble, S., & Scheinost, D. (2020). The Constrained Network-Based Statistic: A new level of inference for neuroimaging. Medical Image Computing and Computer-Assisted Intervention: MICCAI. In: *International Conference on Medical Image Computing and Computer-Assisted Intervention*, 12267, 458–468. 10.1007/978-3-030-59728-3_45 PMC8052680 33870336

[34] Greicius, M. D., Krasnow, B., Reiss, A. L. & Menon, V. Functional connectivity in the resting brain: a network analysis of the default mode hypothesis. *Proc. Natl. Acad. Sci. U.S.A.* **100** (1), 253–258 (2003). 12506194 10.1073/pnas.0135058100 PMC140943

[35] Spreng, R. N. The fallacy of a task-negative network. *Front. Psychol.* **3**, 145 (2012). 22593750 10.3389/fpsyg.2012.00145 PMC3349953

[36] Chou, T., Deckersbach, T., Dougherty, D. D. & Hooley, J. M. The default mode network and rumination in individuals at risk for depression. *Social Cogn. Affect. Neurosci.* **18** (1), nsad032 (2023). 10.1093/scan/nsad032 PMC10634292 37261927

[37] Fan, F. et al. Development of the default-mode network during childhood and adolescence: A longitudinal resting-state fMRI study. *NeuroImage* **226** (117581), 117581 (2021). 33221440 10.1016/j.neuroimage.2020.117581

[38] Langner, R. & Eickhoff, S. B. Sustaining attention to simple tasks: a meta-analytic review of the neural mechanisms of vigilant attention. *Psychol. Bull.* **139** (4), 870–900 (2013). 23163491 10.1037/a0030694 PMC3627747

[39] Sylvester, C. M. et al. Functional network dysfunction in anxiety and anxiety disorders. *Trends Neurosci.* **35** (9), 527–535 (2012). 22658924 10.1016/j.tins.2012.04.012 PMC3432139

[40] Geng, H., Li, X., Chen, J., Li, X. & Gu, R. Decreased intra- and inter-salience network functional connectivity is related to trait anxiety in adolescents. *Front. Behav. Neurosci.* **9**, 350 (2015). 26834594 10.3389/fnbeh.2015.00350 PMC4720749

[41] Marek, S. et al. Reproducible brain-wide association studies require thousands of individuals. *Nature* **603** (7902), 654–660 (2022). 35296861 10.1038/s41586-022-04492-9 PMC8991999

[42] Shechner, T. et al. Empirical examination of the potential adverse psychological effects associated with pediatric FMRI scanning. *J. Child Adolesc. Psychopharmacol.* **23** (5), 357–362 (2013). 23738869 10.1089/cap.2012.0076 PMC3689936

[43] Elwood, L. S., Wolitzky-Taylor, K. & Olatunji, B. O. Measurement of anxious traits: a contemporary review and synthesis. *Anxiety Stress Coping*. **25** (6), 647–666 (2012). 21644113 10.1080/10615806.2011.582949

[44] Knowles, K. A. & Olatunji, B. O. Specificity of trait anxiety in anxiety and depression: Meta-analysis of the State-Trait anxiety inventory. *Clin. Psychol. Rev.* **82** (101928), 101928 (2020). 33091745 10.1016/j.cpr.2020.101928 PMC7680410

[45] Penninx, B. W., Pine, D. S., Holmes, E. A. & Reif, A. Anxiety disorders. *Lancet* **397** (10277), 914–927 (2021). 33581801 10.1016/S0140-6736(21)00359-7 PMC9248771

[46] Menon, V. Large-scale brain networks and psychopathology: a unifying triple network model. *Trends Cogn. Sci.* **15** (10), 483–506 (2011). 21908230 10.1016/j.tics.2011.08.003

[47] Henze, G. I. et al. The ups and downs of brain stress: extending the triple network hypothesis. *Biol. Psychiatry: Cogn. Neurosci. Neuroimaging* (2025). 10.1016/j.bpsc.2025.08.004 40850355 10.1016/j.bpsc.2025.08.004

[48] Wu, K. C. et al. Increasing diversity in neuroimaging research: Participant-driven recommendations from a qualitative study of an under-represented sample. *Dev. Cogn. Neurosci.* **70** (101474), 101474 (2024). 39541798 10.1016/j.dcn.2024.101474 PMC11609318

[49] Michalska, K. J. & Díaz, D. E. A Multi-Component model of emotion response convergence: implications for the development of psychopathology. *Emot. Rev.* **17** (4), 247–267 (2025). 40979676 10.1177/17540739251335577 PMC12445727

[50] Waskom, M. L. Seaborn: statistical data visualization. *J. Open. Source Softw.* **6** (60), 3021 (2021).

