# Supplementary material for: Children’s state anxiety before MRI scanning and resting state functional connectivity in large scale brain networks
Source: Sci Rep. 2026 Jan 16;16:4383. doi: 10.1038/s41598-025-34410-8 (PMC12865014; doi:10.1038/s41598-025-34410-8)
Supplement: Supplementary file 1 — Supplementary Material 1 [file 41598_2025_34410_MOESM1_ESM.docx]

**Supplement 1**


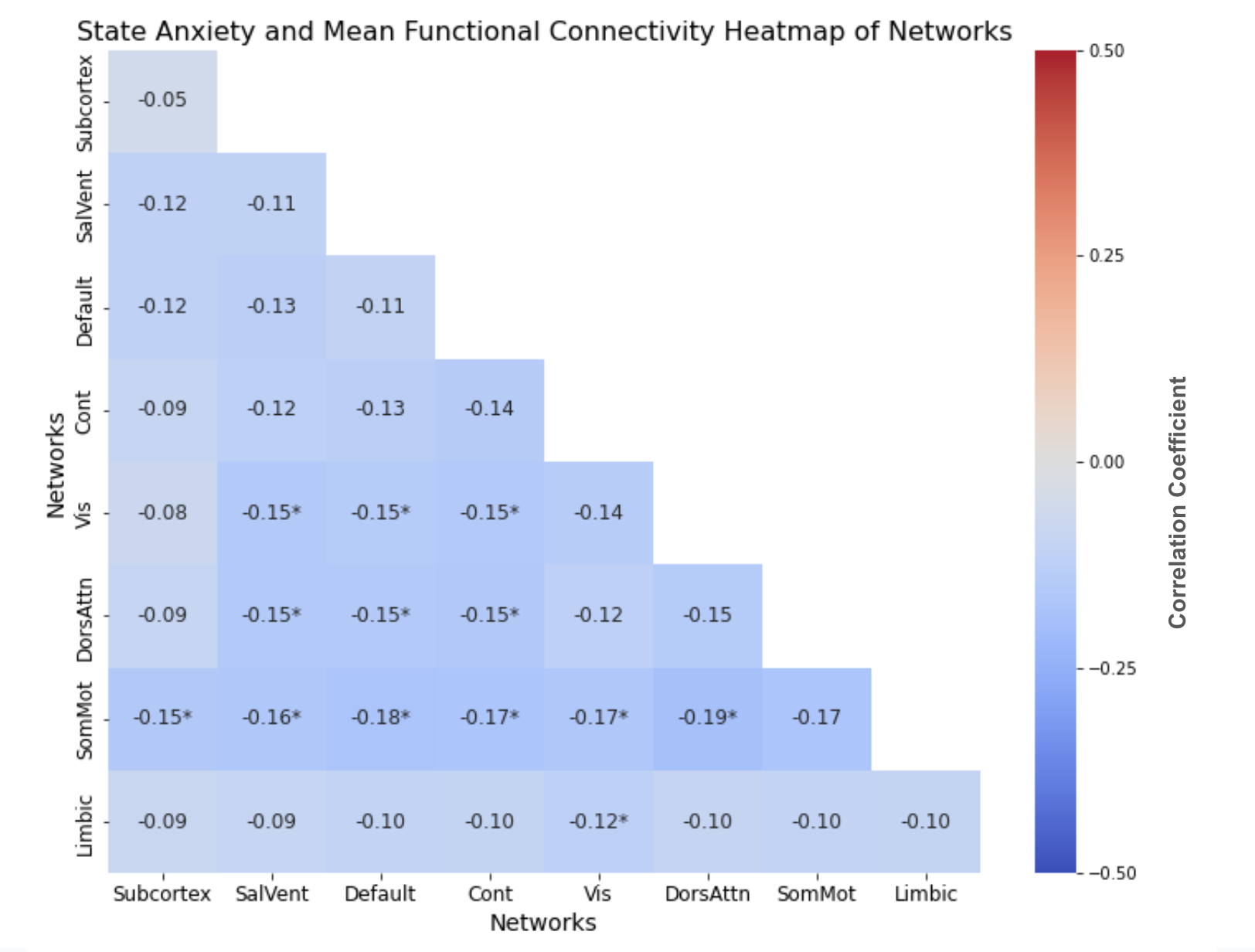


**Supplement 1. NIMH and UCR Sample Resting-State Functional Connectivity.** The heat map displays partial correlation between pre-scan state anxiety and resting-state functional connectivity (rs-FC) within and between 7 networks, controlling for age, trait anxiety, site, and scanner. No significant associations passed corrections (all *p*s > .00139). Red color represents positive connectivity between networks, whereas blue represents negative connectivity. Subcortex = Subcortical Regions, SalVent = Salience Ventral Attention Network, Default = Default Mode Network, Cont = Control Network, Vis = Visual Network, DorsAttn = Dorsal Attention Network, SomMot = Somatomotor Network, Limbic = Limbic Network. Heatmap was generated using Python’s Seaborn data visualization package (version 0.13.2.; <https://seaborn.pydata.org>).^[50]^

**Supplement 2**

**

**

**Supplement 2. NIMH and UCR Sample Network Connectivity P-Values.** Heat maps **A** and **C** display p-values of the partial correlations between pre-scan state anxiety and resting-state functional connectivity (rs-FC) within and between 7 networks, controlling for age, trait anxiety, and scanner. Heat maps **B** and **D** display p-values of the partial correlations between pre-scan state anxiety and resting-state functional connectivity (rs-FC) within and between 7 networks, controlling for only age and scanner. Orange color represents a p-value closer to 1.00, whereas blue represents a p-value closer to 0.00. Subcortex = Subcortical Regions, SalVent = Salience Ventral Attention Network, Default = Default Mode Network, Cont = Control Network, Vis = Visual Network, DorsAttn = Dorsal Attention Network, SomMot = Somatomotor Network, Limbic = Limbic Network. Heatmap was generated using Python’s Seaborn data visualization package (version 0.13.2.; <https://seaborn.pydata.org>).^[50]^
